# Dynamic Observation of the Effect of Maternal Caries on the Oral Microbiota of Infants Aged 12–24 Months

**DOI:** 10.3389/fcimb.2021.637394

**Published:** 2021-05-21

**Authors:** Fei Li, Ding Fu, Danying Tao, Xiping Feng, May Chun Mei Wong, Wei Xu, Haixia Lu

**Affiliations:** ^1^ Department of Preventive Dentistry, Shanghai Ninth People’s Hospital, Shanghai Jiao Tong University School of Medicine, College of Stomatology, Shanghai Jiao Tong University, National Center for Stomatology, National Clinical Research Center for Oral Diseases, Shanghai Key Laboratory of Stomatology, Shanghai, China; ^2^ Department of Orthodontics, Hangzhou Stomatology Hospital, Hangzhou, China; ^3^ Dental Public Health, Faculty of Dentistry, University of Hong Kong, Hong Kong, Hong Kong; ^4^ Department of Pediatric Dentistry, Shanghai Stomatological Hospital, Oral Biomedical Engineering Laboratory, Shanghai Stomatological Hospital, Fudan University, Shanghai, China

**Keywords:** dental caries, oral microbiota, 16S rRNA gene sequencing, early childhood caries, supragingival plaque

## Abstract

**Aim:**

To provide a dynamic description of the oral microbial composition in mothers with and without dental caries and their children aging 12-24 months.

**Methodology:**

A total of 20 pairs of mothers and their children aged 12 months were included and followed up at 18 and 24 months of age. Ten mothers with dental caries(MEG) and their children(CEG) were in the exposure group, and ten caries-free mothers(MCG) and their children(CCG)in control group. Supragingival plaque biofilm samples were collected and DNA was extracted for bacterial 16S rRNA gene sequencing.

**Results:**

A total of 18 pairs completed follow-ups. At a 3% divergence level, the number of common operational taxonomic units found between the mothers and children increased as the children aged. *Proteobacteria*, *Bacteroidetes*, *Firmicutes*, *Fusobacteria*, and *Actinobacteria* accounted for more than 80% phyla of each group. A microbial community structure analysis showed that the differences between mothers and children were significant in all groups except for the MEG24 and CEG24 groups.

**Conclusions:**

Oral microbiota of children was more like their mothers’ with increasing age, regardless of whether the mothers had dental caries. Mothers with dental caries may have a greater influence on the oral microbiota of children’s than those without dental caries as children age.

## Introduction

Dental caries is the most common chronic disease of childhood (Islam et al., 2007). Early childhood caries (ECC) not only impair the oral health of children, but also affect the general growth and cognitive abilities of children as they age (Martinsjunior et al., 2013). Although largely preventable, ECC remains the most common chronic childhood disease, accounting for nearly 1.8 billion new cases globally each year (Vos et al., 2017). Furthermore, according to the 4^th^ National Oral Health Survey in China, the prevalence of dental caries has reached 71.9% among 5-year-old children (Hurley et al., 2019), suggesting that ECC is a significant public health problem in China. Thus, early prevention and intervention at the initial stage of ECC is essential.

The oral cavity harbors more than 700 bacterial taxa (Paster et al., 2006), and it has been reported that dental caries involves interactions between the tooth structure and the microbial biofilm formed on the tooth surface (Pitts et al., 2017). Actually, the oral cavity of newborn babies is usually sterile or contains only small amounts of bacteria that are transmitted from their mother´s vagina during delivery (Carlsson and Gothefors, 1975). Moreover, it has been demonstrated that microorganisms can be vertically transmitted from mother to infant (Alves et al., 2009). And the salivary microbiome is dynamic during the first 2 year of life, and is influenced by whether the child was breastfed, and is associated with maternal oral health status (Ramadugu et al., 2021). One study has reported that the establishment and persistence of normal oral flora in children are closely associated with the local oral flora of their parents (Filoche et al., 2010). Therefore, in any study of the cariogenic mechanisms of microorganisms, the maternal influence is not negligible.

As vertically transmission have long been suggested to play a major role in the spread of cariogenic bacteria, most of these studies focused on specific species, including mutans streptococci and lactobacilli (Teanpaisan et al., 2012; Subramaniam and Suresh, 2019). However, recent advances in molecular approaches have enabled scientists to better understand the mother-to-child transmission from microecology sight using technique such as16S rRNA gene amplicon sequencing (Nyvad et al., 2013). Additionally, there is insufficient scientific evidence regarding the dynamic change of oral microbial diversity from the eruption of deciduous teeth to the emergence of ECC and whether this change is closely related to the dental caries level of the mother. Therefore, in this study, based on the same participants in our previous study (Li et al., 2018), we aimed to provide a dynamic description of the oral microbial composition of mothers and their children as they aged from 12 to 18 and then to 24 months using high-throughput sequencing techniques. Additionally, we aimed to compare the oral microbial diversity of mothers with or without dental caries to their children as they aged from 12 to 24 months.

## Materials and Methods

### Study Participants

The study participants were 12-month-old children and their mothers. From October 2012 to March 2013, the socioeconomic status and oral health-related quality of life of pregnant women was investigated in three maternal and childcare service centers at the county level in Shanghai. The children were followed up at 12, 18, and 24 months of age. According to the mothers’ dental caries status when the children were 12 months old, the pairs were placed into one of two groups: mothers with dental caries (MEG) and their children (CEG), and mothers without dental caries (MCG) and their children (CCG). The inclusion criteria for the exposure group were as follows: (1) mothers that had participated in the previous project; (2) mothers with one or more untreated dental caries, and without missing or filling teeth when their infants were12 months old; (3) healthy infants without hereditary diseases or deformities; (4) existing tooth eruption in the infant; (5) mothers and their infants without intake of antibiotics in the previous 2 months; and (6) infants without prior history of dental treatments. The inclusion criteria for those in the control group were the same as for those in the exposure group except the mothers had to be caries-free when the infants were 12, 18, and 24 months old (same criteria for 1 and 3-6).

The sample size calculation was as following: the main outcome variable of the present study is the Shannon index when children were followed up to 24 months, because the Shannon index is the most commonly used index to assess microbial diversity. Through literature review and pilot study, it is assumed that the standard deviation of the Shannon index is 1.1, and the mean difference of Shannon index between the exposed group and the control group is 0.6 in a two-sided 5% significance level test with 80% power, each group requires 6 children. According to the two-year loss to follow-up rate is 20%, each group requires at least 8 children finally. A total of 20 pairs of children (aged 12 months) and mothers were included in this study. The study was conducted in accordance with the Declaration of Helsinki, and the protocol was approved by the Ethics Committee of the Ninth People’s Hospital, Shanghai Jiao Tong University (Project identification code: 201410).Written informed consent was obtained from all the parents of the children.

### Oral Examination

For the clinical oral examination of the mothers and children (at 12, 18, and 24 months of age), the participants’ dental caries and oral hygiene statuses were assessed. The mothers’ dental caries statuses were diagnosed by a trained and licensed dentist according to the criteria recommended by the World Health Organization (WHO) (Organization, 2013). Dental caries detected visually at the cavitation level was recorded using a mouth-mirror and a lightweight community periodontal index probe under artificial light, and early caries was not recorded. All teeth present in the mouth (including third molars) were subjected to clinical oral examination. For the dental caries of infants at 12, 18, and 24 months of age, the clinical manifestation of early caries was defined as a surface with opacities and discoloration when viewed as wet, while caries with a cavity were defined according to the WHO recommendations. The oral hygiene status of both the mothers and infants were evaluated using the visible plaque index (VPI) and scored as either 0 or 1, which respectively corresponded to the absence or the presence of dental plaque on two surfaces per tooth. The percentage of the surfaces with plaque was calculated and ranged from 0 to 100%.

### Sample Collection

The dental plaque biofilms were collected to extract genomic DNA. After fasting for 2h, supragingival plaques were sampled from all the mothers’ and children’ teeth at 12, 18, and 24 months of age using a sterile curettage. Pooled plaque samples were immediately placed in a sterile Eppendorf tube with 1 ml of sodium thiosulfate solution, and then preserved at -80°C.

### DNA Isolation, Library Preparation and Sequencing

Total genomic DNA of the dental plaque biofilms were extracted using the QIAGEN QIAamp DNA mini kit (Qiagen, Hilden, Germany). The concentration and purity of the DNA was evaluated using a Nanodrop 2000 ultra-micro spectrophotometer (NanoDrop Technologies, Wilmington, DE, USA). The variable regions (V4-V5) of 16S rDNA were amplified using universal primers 515f (5’-GTGCCAGCMGCCGCGG-3’) and 907Rb (5’-CCGTCAATTCMTTTRAGTTT-3’). About 10-50 ng of DNA was used for PCR-amplification, and the PCR reaction conditions were as follows: initial denaturation at 94°C for 3 min, 35 cycles of denaturation at 94°C for 45s, annealing at 50°C for 1 min, elongation at 72°C for 1.5 min, and a final extension step at 72°C for 10 min. The PCR products were purified using the Gel Extraction Kit (AXYGEN, CA, USA). The quality and the purity of the DNA were evaluated using the Quant-iTPicoGreen dsDNA Assay Kit and the Microplate reader (BioTek, FLx800), respectively. The 16SrRNA library was prepared using the TruSeq Nano DNA LT Library Prep Kit (Illumina) and sequenced with 5% PhiX samples using Illumina MiSeq sequencers with 600 cycles.

### Data Preprocessing

Quality control was performed on the raw data in the FASTQ format by removing the reads with an average quality of < Q20, a length < 150 bp, and chimera and ambiguous bases. Two high-quality paired reads were recombined using the FLASH software (version 1.2.7) (Magoč and Salzberg, 2011) to obtain the effective sequences.

### OTU Clustering and Annotation

The operational taxonomic units (OTUs) with 97% similarity were identified and classified using the taxonomy classification methods in the UCLUST software (Edgar, 2010). The longest sequence in each OTU was selected as the representative sequence. An OTU table was constructed, and OTUs with <0.00001 abundance were filtered (Bokulich et al., 2013). The representative sequence for each OTU was annotated using the ribosomal database project classifier (version 2.2) (Quast et al., 2012) based on the Silva Database (Wang et al., 2007). A taxonomic table was obtained for each sample.

### Alpha Diversity Analysis

Rarefaction curves were used to determine the sequencing depth, species accumulation curves were used to determine whether the sample size was sufficient, and rank abundance curves were used to assess the microbial diversity of the samples. The complexity of species diversity was analyzed using alpha diversity *via* the Chao1, ACE, Shannon, and Simpson indices. Chao1 and ACE were used to estimate the community richness (Lee, 1992; Yang, 1993), while the Shannon and Simpson indices (Shannon, 1948; Simpson, 1949) were used to estimate community diversity.

### Community Composition and Beta Diversity Analyses

A community composition analysis was performed to statistically compare the OTU results with community structure databases at the phylum and genus levels using QIIME (Version 1.9.0, University of Colorado, Boulder USA) (Altschul, 2012). An analysis of variance with multiple comparisons was used to compare the dominant bacterial genera of the different groups. A cluster analysis using R software was performed on the abundance information of the top 50 genera with the highest abundance. Beta diversity was analyzed for taxonomic classification and species abundance at the genus level through a principal components analysis (PCA). The PCA plot was generated using the R vegan package (version 3.1.2, University of Auckland, New Zealand).

### Statistical Analysis

Differences of questionnaire and oral clinical examination data between two groups were assessed by Mann–Whitney test and Bonferroni adjustments were used for multiple testing. One-way permutational multivariate analysis of variance (PERMANOVA) was calculated in QIIME software. All statistical analysis were produced using the R package. A P < 0.05 was considered to be significant.

## Results

### Sample Characteristics

A total of20 pairs of mothers and their children aged 12 months were enrolled in this study. One pair was lost to follow-up at 18 months and one at 24 months (both children and their mother without dental caries) due to the nonlocal residence change. Therefore, 18 pairs completed all three follow-ups. As shown in **Tables S1** and **S2**, there was no significant difference in the social demographic background or oral health-related behaviors between the exposed group and the non-exposed group (all P > 0.05).

At 12 months of age, all children had eruptions of only deciduous anterior teeth (mean number of erupted teeth: 5.6; mean VPI: 0.11 ± 0.18). At 18 months of age, one child in CEG group had developed white spot lesions on two teeth (mean number of erupted teeth: 11.2; mean VPI: 0.19 ± 0.15). At 24 months of age, two children in CEG group had developed white spot lesions on three teeth (mean number of erupted teeth: 15.6; mean VPI: 0.18 ± 0.12). During the follow-ups, 54 samples of supragingival plaque biofilms were obtained from the mothers and their children.

### Distribution of OTUs

**Figure 1:**
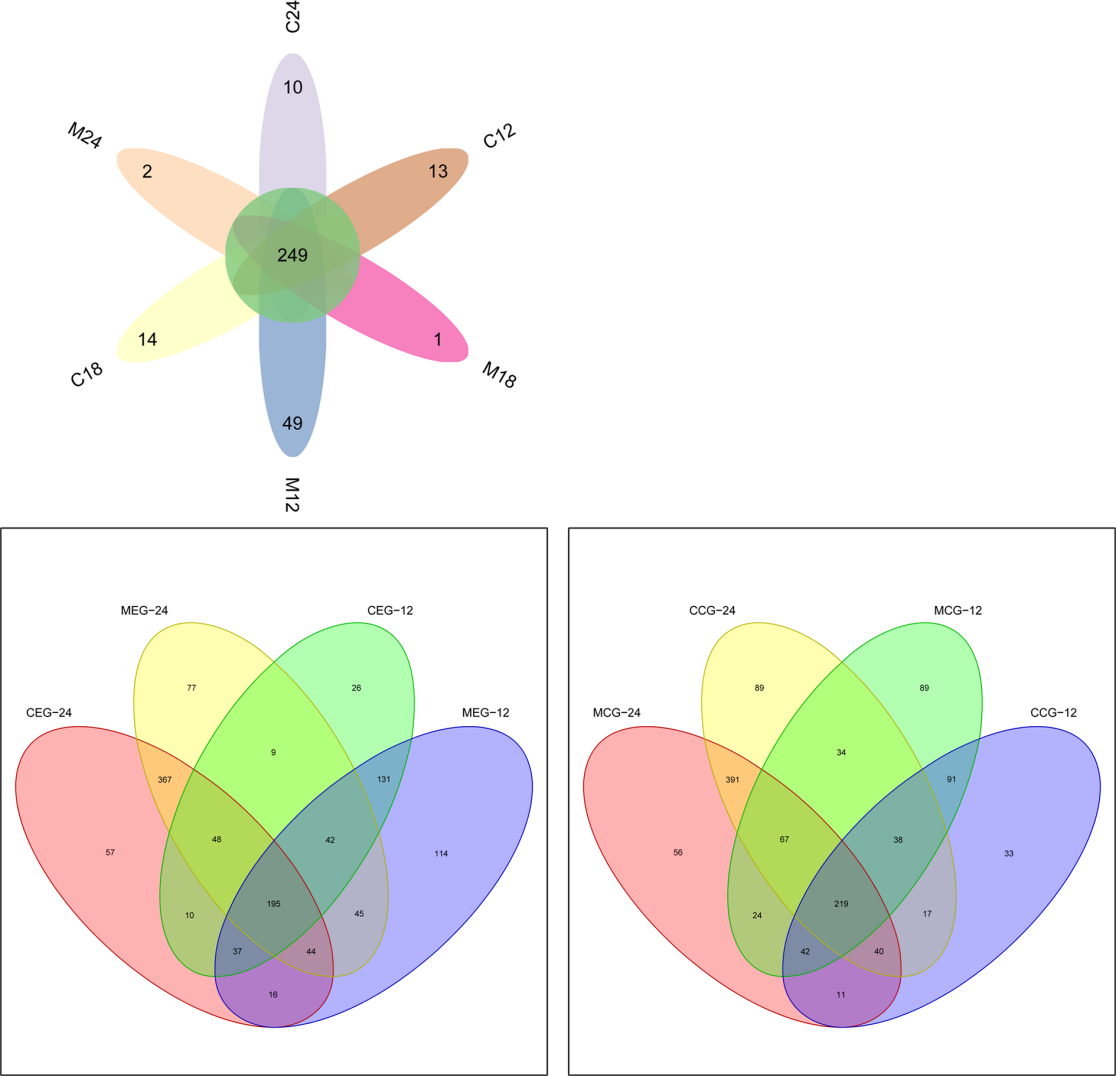
Venn diagram showing shared and unique OTUs at 97% identity between the mother and child groups.

OTU clustering and sequence annotation were conducted based on the sequences at a 3% dissimilarity level, and the OTU tables obtained were used for subsequent analyses. The OTU distributions for mothers (M12, M18, and M24) and children (C12, C18, and C24) at 12, 18, and 24 months, respectively, are shown in **Figure 1**. There were 516, 725, and 817 OTUs in common between M12 and C12, M18 and C18, and M24 and C24, respectively. Additionally, at 12 months and 24 months of age, a total of 405 and 654 OTUs were identified in common between the MEG and CEG, and a total of 390 and 717 OTUs were identified in common between the MCG and CCG, respectively. These data showed that the number of common OTUs found between the mothers and children increased as the children aged, indicating oral microbiota of children was more like their mothers’ with increasing age, regardless of whether the mothers had dental caries.

### OTU Abundance Analysis

**Figure 2:**
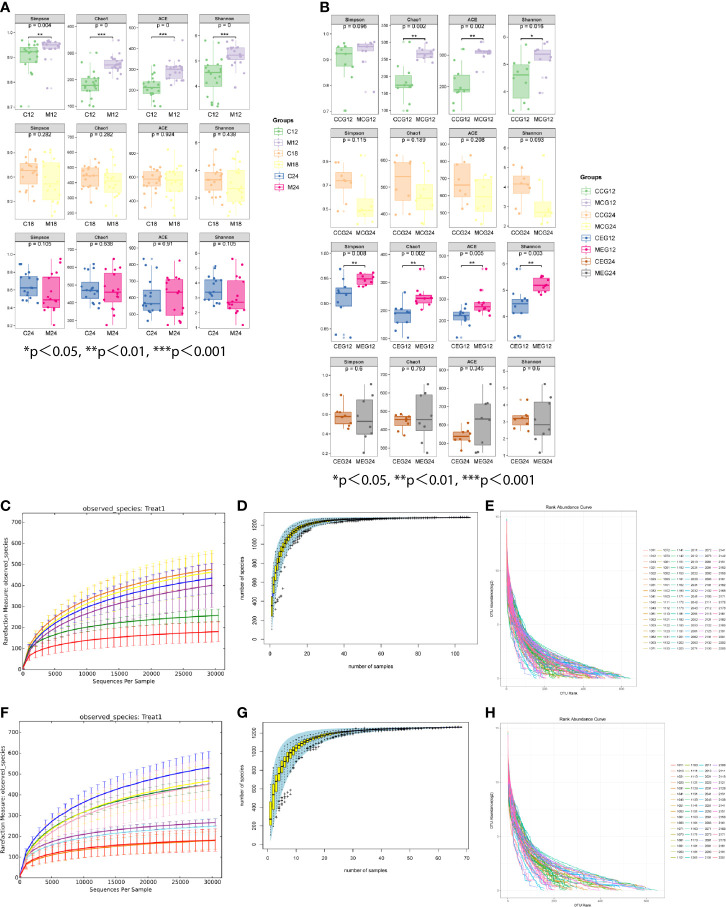
The alpha diversity indices of different groups. **(A)** The alpha diversity indices (Chao1, Shannon, Simpson, and ACE) between mother (M) and child (C) at 12, 18, and 24 months of age. **(B)** The alpha diversity indices (Chao1, Shannon, Simpson, and ACE) between M and C in the exposure group (MEG and CEG) and the control group (MCG and CCG) at 12 and 24 months of age. The rarefaction curves **(C, F)**, species accumulation curves **(D, G)** and abundance distribution curves **(E, H)** for OTUs.

The alpha diversity indices, which included Chao1, Shannon, Simpson, and ACE, are shown in **Figures 2A, B**. The results demonstrated that in M12 the indices were significantly higher than those in C12, but there were no significant differences between M18 and C18 or between M24 and C24.Additionally, the Chao1, Shannon, and ACE in the MCG12 were significantly higher than those in the CCG12. The alpha diversity indices in the MEG12 were significantly higher than those in the CEG12. No significant differences were identified between the MCG24 and CCG24 or between the MEG24 and CEG24 for all four alpha diversity indices. As for the diversity difference between CEG and CCG, significant differences were identified only at M24, but not at M12 and M18 (**Table S3**). The rarefaction curve showed that each group tended to flatten, and increasing sequencing depths did not help to reveal new OTUs, indicating that the sequencing results reasonably reflected the microorganisms present in the samples (**Figures 2C, F**). The species accumulation curves indicated that the sequencing depth in this study was also adequate (**Figures 2D, G**). The abundance distribution curves showed that the oral microbial diversity in each sample was mainly composed of a few microorganisms, while other microorganisms were present at low rates (**Figures 2E, H**).

### Microbial Community Structure Analysis

**Figure 3:**
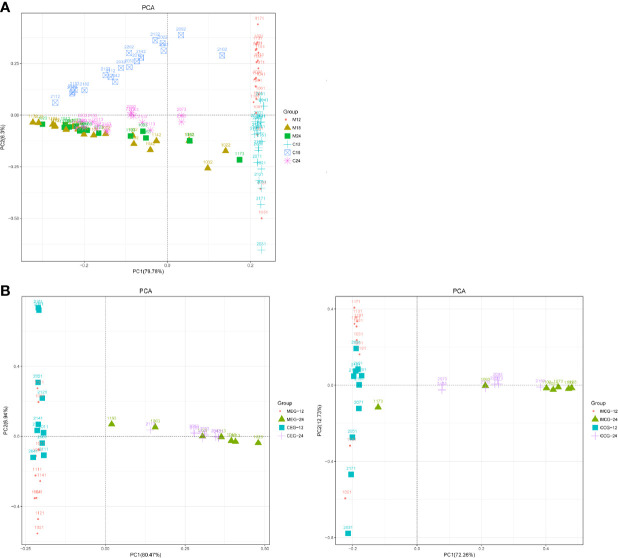
PCA based on the UniFrac distances at the OTU level at 97% identity for **(A)** mother (M) and child (C) at 12, 18, and 24 months of age; **(B)** M or C in the exposure group (MEG or CEG) and the control group (MCG or CCG) at 12 and 24 months of age.

To analyze the microbial community structure of all the samples, a PCA was performed at the genus level, and the results were further compared between the groups through an Adonis/PERMANOVA analysis. The PCA showed that most of the samples in each group were clustered, and that most samples in M18, M24 and C24,the MEG24 and CEG24, and the MCG24 and CCG24 were clustered together (**Figure 3**). The results of the Adonis/PERMANOVA analysis showed that the differences between M12 and C12 (F = 10.735, p = 0.001), between M18 and C18 (F = 7.5753, p = 0.001), and between M24 and C24 (F = 5.7414, p = 0.001) were all statistically significant. Additionally, the differences between MEG12 and CEG12 (F = 4.581, p = 0.001), MCG12 and CCG12 (F = 7.1395, p = 0.001), and MCG24 and CCG24 (F = 3.2081, p = 0.001) were significant, while the differences between MEG24 and CEG24 (F = 1.7912, p = 0.152) were not significant, suggesting mothers with dental caries may have a greater influence on the oral microbiota of children’s than those without dental caries as children age.

### Microbial Composition Analysis

**Figure 4:**
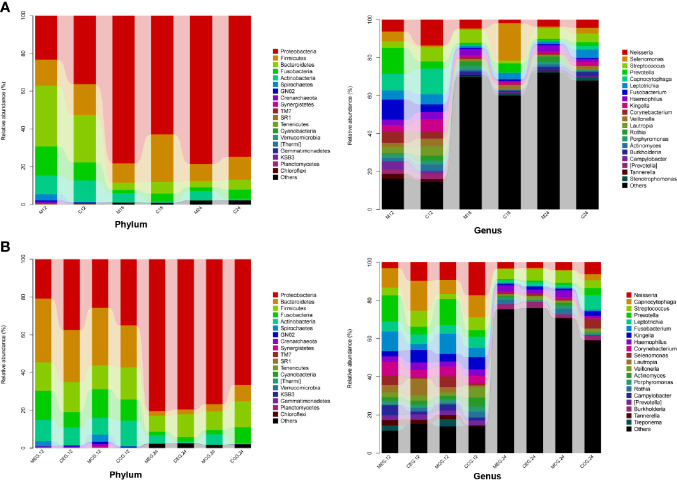
Classification and compositional ratios of the different groups sequenced at the taxonomic level. **(A)** mother (M) and child (C) at 12, 18, and 24 months of age; **(B)** M or C in the exposure group (MEG or CEG) and the control group (MCG or CCG) at 12 and 24 months of age.

At the taxonomic level, the microbial compositional ratios of the mothers and children at the three time points are shown in **Figure 4A**. *Proteobacteria*, *Bacteroidetes*, *Firmicutes*, *Fusobacteria*, and *Actinobacteria* were the dominant bacterial phyla, accounting for more than 80% of each group. The top six dominant genera in M12 were *Prevotella*, *Fusobacterium*, *Capnocytophaga, Neisseria, Corynebacterium, and Selenomonas*; the top six in C12 were *Capnocytophaga*, *Neisseria*, *Streptococcus, Kingella, Leptotrichia, and Lautropia*; that in M18 were *Streptococcus*, *Neisseria, Haemophilus, Rothia, Burkholderia, and Porphyromonas*; that in C18 were *Selenomonas*, *Prevotella, Leptotrichia, Veillonella, Neisseria, and Streptococcus*; that in M24 were *Streptococcus*, *Neisseria*, *Haemophilus, Rothia, Burkholderia, and Porphyromonas*; and that in C24 were *Streptococcus*, *Leptotrichia*, *Neisseria, Selenomonas, Burkholderia, and Prevotella.*

In addition, the microbial relative abundance in the MEG and CEG at 12 months and 24 months are shown in **Figure 4B**. Similarly, *Proteobacteria*, *Bacteroidetes*, *Firmicutes*, *Fusobacteria*, and *Actinobacteria* were the dominant phyla. The top three dominant genera in the MEG12 group were *Prevotella*, *Fusobacterium*, Corynebacterium, Leptotrichia, and Campylobacter; that in the CEG12 group were Capnocytophaga, Neisseria, Lautropia, Streptococcus, Kingella, and Leptotrichia; that in the MCG12 group were Prevotella, Fusobacterium, Neisseria, Capnocytophaga, Selenomonas, and Corynebacterium; that in the CCG12 group were Neisseria, Capnocytophaga, Streptococcus, Kingella, Leptotrichia, and Veillonella; that in the MEG24 group were Streptococcus, Neisseria, Haemophilus, Burkholderia, Rothia, and Leptotrichia; that in the CEG24 group were Streptococcus, Burkholderia, Neisseria, Leptotrichia, Haemophilus, and Veillonella; that in the MCG24 group were Streptococcus, Neisseria, Haemophilus, Rothia, Burkholderia, and Porphyromonas; and that in the CCG24 group were Leptotrichia, Neisseria, Selenomonas, Streptococcus, Prevotella, and Capnocytophaga.

**Figure 5:**
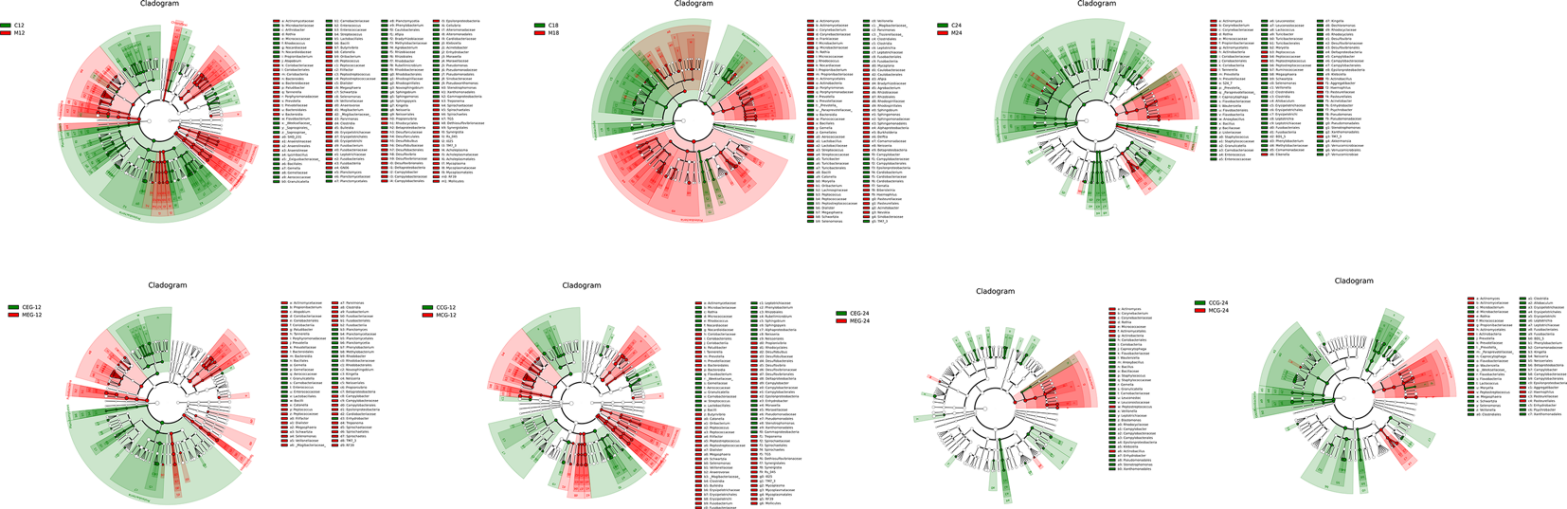
The linear discriminant analysis effect size (LefSe). The colored nodes from the inner to outer circles represent the top abundant taxa from phylum to genus level. The white nodes represent taxa that are not significantly different between the groups, while nodes with other colors represent taxa that are significantly different between the groups.

The linear discriminant analysis effect size (LefSe) method was used to compare the enriched taxa in each group (**Figure 5**). In the 12-month group, mothers tended to have a higher relative abundance of *Bacteroidia*, *Prevotellaceae*, and *Prevotella*, while the children tended to have a higher relative abundance of *Proteobacteria*, *Betaproteobacteria*, and *Neisseriales*. In the 18-month group, mothers had a higher relative abundance of *Proteobacteria*, *Bacilli*, and *Streptococcus*, while the children had a higher relative abundance of *Clostridia*, *Selenomonas*, and *Firmicutes*. In the 24-month group, mothers had a higher relative abundance of *Actinomycetales*, *Actinobacteria*, and *Rothia*, while children had a higher relative abundance of *Clostridia*, *Firmicutes*, and *Leptotrichia*.

In the MEG12, there was a higher relative abundance of *Bacteroidia*, *Prevotellaceae*, and *Prevotella* and in the CEG12,*Proteobacteria*, *Betaproteobacteria*, and *Neisseriales* were relatively more abundant. In the MCG12, *Bacteroidia*, *Prevotellaceae*, and *Prevotella* were relatively more abundant and in the CCG12, *Neisseriales*, *Neisseria*, and *Bacilli* had a higher relative abundance. Moreover, *Actinomycetales*, *Actinobacteria*, and *Micrococcaceae* were relatively more abundant in the MEG24, while *Veillonella*, *Xanthomonadales*, and *Stenotrophomonas* had a higher relative abundance in the CEG24. There was a higher relative abundance of *Actinomycetales*, *Actinobacteria*, and *Rothia* in the MCG24 and *Fusobacteriales*, *Fusobacteria*, and *Leptotrichia* had a higher relative abundance in CCG24. Notably, there were fewer differences in taxa between the MEG24 and CEG24 compared to that between the other three comparison groups. Additionally, the children had more enriched taxa than the mothers when the children were 24 months old.

## Discussion

Dental caries is a complex multifactorial disease. A recent heritability study based on larger families reported that the heritability of dental caries in primary dentition is more than 50% (Wang et al., 2010). Additionally, environmental determinants play a key role in the susceptibility of children to caries (Lukacs and Largaespada, 2006), and mothers are an important environmental factor associated with ECC (Priyadarshini et al., 2013). Our previous study demonstrated that maternal oral microbial diversity has an overall effect on 12-month-old infants (Tao et al., 2018). In the present study, we compared the oral microbial diversityof1) mothers to that of their children at 12, 18, and 24 months of age and 2) mothers with or without dental caries to that of their children at 12 and 24 months of age.

At a 3% divergence level, there were 516, 725, and 817 OTUs in common between mothers and children at 12, 18, and 24 months of age, respectively. Additionally, 405 and 654 OTUs were identified in common between the MEG and CEG at 12 months and 24 months, respectively. A total of 390 and 717 OTUs were obtained in common between the MCG and CCG at 12 months and 24 months, respectively. The number of common OTUs increased with time, indicating that the children’s oral microbiota were more similar to their mothers with increasing age, regardless of whether the mothers had dental caries. It has been reported that an infant’s immune system is immature, and the plaque ecosystem evolves and matures gradually (Tao et al., 2018), which may explain the increase in microbial diversity with age. Additionally, Cephas et al. (2011) have reported that although the detection rate of oral microorganisms in infants is low, their oral microbial diversity is greater than that of their parents. Similarly, in our study, the LefSe analysis showed that mothers had less enriched taxa compared to their children at 24 months of age.

The oral microbial diversity in each group was compared using alpha diversity indices, including Chao1, Shannon, Simpson, and ACE. Comparison results showed that the alpha diversity indices in the mother groups were significantly higher than that in the children groups at 12 months, but there were no significant differences between the mothers and children at 18 and 24 months. Similar results were found in the mothers and children with or without caries, namely, the alpha diversity indices in the MEG12 were significantly higher than that in the CEG12.Additionally, the Chao1, Shannon, and ACE in the MCG12 were significantly higher than that in the CCG12. Moreover, the overall similarity between microbial communities in each group was determined by PCA. The results showed that the community composition in most of the children’s samples differed from that of the mothers at 12 months regardless of whether the mothers had caries. Taken together, these results indicated that alpha diversity indices and PCA analyses were consistent when assessing for microbial diversity. The oral microbiota of the children was more similar to that of their mothers with increasing age, regardless of whether the mothers had dental caries.

Mother, who generally play as the main caregivers, would exhibit primary influence in the formation, transmission, and environmental conservation and social development of her children (Silva and De Sousa, 2000). Given this, there will be inevitable connection between the oral health of both the mother and their child. It has been reported that feeding practices are considered to be risk factors for ECC (Hooley et al., 2012). Even caregiver behavior to prevent vertical transmission was not effective in reducing levels of childhood caries (Wakaguri et al., 2011). In terms of the present research, the children’s oral microbiota were found to be more similar to their mothers after we had adjusted the sociodemographic background and oral health related behavior of mothers between two groups. This might be also attributed to the maternal sharing of utensils or close contact during child feeding. Additionally, given that mothers with dental caries were likely to affect their children, mothers should be the target of choice for intervention to prevent early childhood caries, and active treatment of dental caries should be performed during pregnancy.

The human species includes a large diversity of microbiomes, including about 10,000 different species of microorganisms, which are distributed into 16 phyla, 4 of which are closely related to oral health: *Actinobacteria*, *Bacteroidetes*, *Firmicutes*, and *Proteobacteria* (Zaura et al., 2009; Zarco et al., 2012; Simpson and Thomas, 2016). In our study, the composition of microorganisms among the different groups was similar. *Proteobacteria*, *Bacteroidetes*, *Firmicutes*, *Fusobacteria*, and *Actinobacteria* were the dominant bacterial phyla, accounting for more than 80% of each group, which was consistent with findings in the aforementioned studies. *Proteobacteria* are present at various body sites, such as the oral cavity, skin, and tongue (Huttenhower et al., 2012). *Bacteroidetes* make up a large portion of the natural intestinal microbiota. *Tannerella forsythia*, belonging to *Bacteroidetes*, is associated with oral infections such as periodontal disease (Socransky et al., 1998). *Fusobacterium nucleatum*, a member of *Fusobacteria*, is a resident member of the human oral cavity and plays an important role in the development of periodontal disease (Socransky et al., 1998).

A microbial community structure analysis showed that the differences between mothers and children were all significant except for those between mothers with dental caries and their children at 24 months of age (F = 1.7912, p = 0.152). Additionally, there were fewer differences in taxa between the MEG24 and CEG24 compared with that between the other three comparison groups. These findings suggest that mothers with dental caries may have a greater influence on their children’s oral microbial communities than those without dental caries as the children age. Studies have reported that a vertical transmission of cariogenic bacteria does exist between mother and child (Caufield et al., 1988; Law et al., 2007: Alves et al., 2009). Importantly, a recent study by (Leong et al., 2013) revealed that in the pre-dentate stage, infants can be colonized with cariogenic bacteria. Given that, our study suggest that vertical transmission of cariogenic bacteria may happen at the microbiome level, which means that infants might inherit the microbial imbalance from their mother with dental caries due to the intimate contact. The susceptibility to dental caries would be then established once the cariogenic microorganisms colonized as the core microbiome in the cavity of infants. This assumption would be consistent with the ecological plaque theory that indigenous microbiota can drive dental caries in a determined environment.

In this study, *Prevotella*, *Fusobacterium*, *Capnocytophaga*, *Streptococcus*, *Neisseria*, and *Haemophilus* were found to be the dominant genera in the MEG groups. Streptococcus, Neisseria, Porphyromonas, and Prevotella have been reported to be strongly associated with dental caries (Aas et al., 2008; Tanner et al., 2011; Nyvad et al., 2013). Additionally, *Fusobacterium* and *Prevotella* were identified as potential contributors to ECC because their abundance was altered in the caries microbiota (Almeida et al., 2000; Obata et al., 2014; Xu et al., 2014). In the CEG group, *Neisseria, Capnocytophaga*, *Lautropia*, *Haemophilus*, *Burkholderia*, and *Streptococcus* were the dominant genera, 4 of which were the same as those in the MEG groups, further suggesting that a mother with dental caries may have an effect on her child developing caries.

Presently, the main limitation of this study was that the follow-up time was too short, and the infants did not present with obvious dental caries by the end of the observation period, which may have influenced the results.

## Conclusion

In conclusion, our study showed that children’s oral microbiota were more similar to that of their mothers with increasing age, regardless of whether the mothers had dental caries. Mothers with dental caries may have a greater influence on the oral microbiota of children’s than those without dental caries as children age.

## Data Availability Statement

The datasets presented in this study can be found in online repositories. The name of the repository and accession number can be found below: National Center for Biotechnology Information (NCBI) BioProject, https://www.ncbi.nlm.nih.gov/bioproject/, PRJNA703092.

## Ethics Statement

The studies involving human participants were reviewed and approved by Ethics Committee of the Ninth People’s Hospital, Shanghai Jiao Tong University (Project identification code: 201410). Written informed consent to participate in this study was provided by the participants’ legal guardian/next of kin.

## Author Contributions

Investigation: DF and DT. Methodology: HL. Project administration: XF. Supervision: MW and HL. Writing - original draft: FL. Writing - review and editing: WX. All authors contributed to the article and approved the submitted version.

## Funding

This study was supported by grants from the General Project of Shanghai Municipal Health Committee (Project No. 201940041), Shanghai Municipal Key Clinical Specialty (Project No. shslczdzk01601), and Shanghai Clinical Research Center for Oral Diseases (19MC1910600).

## Conflict of Interest

The authors declare that the research was conducted in the absence of any commercial or financial relationships that could be construed as a potential conflict of interest.

## References

[B1] Aas J. A. Griffen A. L. Dardis S. R. Lee A. M. Olsen I. Dewhirst F. E.. (2008). Bacteria of Dental Caries in Primary and Permanent Teeth in Children and Young Adults. J. Clin. Microbiol. 46 (4), 1407–1417. 10.1128/JCM.01410-07 18216213 PMC2292933

[B2] Almeida A. G. Roseman M. M. Sheff M. Huntington N. Hughes C. V. (2000). Future Caries Susceptibility in Children With Early Childhood Caries Following Treatment Under General Anesthesia. Pediatr. Dentistry 22 (4), 302–306. 10969437

[B3] Altschul S. F. (2012). Basic Local Alignment Search Tool (BLAST). J. Mol. Biol. 215 (3), 403–410. 10.1016/S0022-2836(05)80360-2 2231712

[B4] Alves A. C. Nogueira R. D. Stipp R. N. Pampolini F. Moraes A. B. Goncalves R. B.. (2009). Prospective Study of Potential Sources of Streptococcus Mutans Transmission in Nursery School Children. J. Med. Microbiol. 58 (4), 476–481. 10.1099/jmm.0.005777-0 19273644

[B5] Bokulich N. Subramanian S. Faith J. Gevers D. Gordon J. I. Knight R.. (2013). Quality-Filtering Vastly Improves Diversity Estimates From Illumina Amplicon Sequencing. Nat. Methods 10 (1), 57–59. 10.1038/nmeth.2276 23202435 PMC3531572

[B6] Carlsson J. Gothefors L. (1975). Transmission of Lactobacillus jensenii and Lactobacillus acidophilus From Mother to Child at Time of Delivery. J. Clin. Microbiol. 1 (2), 124–128. 10.1128/JCM.1.2.124-128.1975 809467 PMC274985

[B7] Caufield P. W. Ratanapridakul K. Allen D. N. Cutter G. (1988). Plasmid-Containing Strains of Streptococcus Mutans Cluster Within Family and Racial Cohorts: Implications for Natural Transmission. Infect. Immun. 56 (12), 3216–3220. 10.1128/IAI.56.12.3216-3220.1988 3182079 PMC259727

[B8] Cephas K. D. Kim J. Mathai R. A. Barry K. A. Dowd S. E. Meline B. S.. (2011). Comparative Analysis of Salivary Bacterial Microbiome Diversity in Edentulous Infants and Their Mothers or Primary Care Givers Using Pyrosequencing. PloS One 6 (8). 10.1371/journal.pone.0023503 PMC3154475 21853142

[B9] Edgar R. (2010). Search and Clustering Orders of Magnitude Faster Than BLAST. Bioinf. (Oxford England) 26 (19), 2460–2461. 10.1093/bioinformatics/btq461 20709691

[B10] Filoche S. Wong L. Sissons C. (2010). Oral Biofilms: Emerging Concepts in Microbial Ecology. J. Dental Res. 89 (1), 8–18. 10.1177/0022034509351812 19918089

[B11] Hooley M. Skouteris H. Boganin C. Satur J. Kilpatrick N. (2012). Parental Influence and the Development of Dental Caries in Children Aged 0-6 Years: A Systematic Review of the Literature. J. Dentistry 40 (11), 873–885. 10.1016/j.jdent.2012.07.013 22842202

[B12] Hurley E. Barrett M. P. Kinirons M. Whelton H. Ryan C. A. Stanton C.. (2019). Comparison of the Salivary and Dentinal Microbiome of Children With Severe-Early Childhood Caries to the Salivary Microbiome of Caries-Free Children. BMC Oral. Health 19 (1), 13. 10.1186/s12903-018-0693-1 30642327 PMC6332856

[B13] Huttenhower C. Gevers D. Knight R. (2012). Structure, Function and Diversity of the Healthy Human Microbiome. Nature 486 (7402), 207–214. 10.1038/nature11234 22699609 PMC3564958

[B14] Islam B. Khan S. N. Khan A. U. (2007). Dental Caries: From Infection to Prevention. Med. Sci. Monit. 13 (11). 10.1051/medsci/200723111063 17968308

[B15] Law V. Seow W. K. Townsend G. C. (2007). Factors Influencing Oral Colonization of Mutans Streptococci in Young Children. Aust. Dental J. 52 (2), 93–100. 10.1111/j.1834-7819.2007.tb00471.x 17687953

[B16] Lee S. M. (1992). Estimating the Number of Classes via Sample Coverage. J. Am. Stat. Assoc. 10.1080/01621459.1992.10475194

[B17] Leong P. M. Gussy M. Barrow S. L. De Silvasanigorski A. Waters E. (2013). A Systematic Review of Risk Factors During First Year of Life for Early Childhood Caries. Int. J. Paediatric Dentistry 23 (4), 235–250. 10.1111/j.1365-263X.2012.01260.x 22925469

[B18] Li F. Tao D. Feng X. Wong M. Mei C. Lu H. (2018). Establishment and Development of Oral Microflora in 12–24 Month-Old Toddlers Monitored by High-Throughput Sequencing. Front. Cell. Infect. Microbiol. 8, 422. 10.3389/fcimb.2018.00422 30564560 PMC6288402

[B19] Lukacs J. R. Largaespada L. L. (2006). Explaining Sex Differences in Dental Caries Prevalence: Saliva, Hormones, and “Life-History” Etiologies. Am. J. Hum. Biol. 18 (4), 540–555. 10.1002/ajhb.20530 16788889

[B20] Magoč T. Salzberg S. (2011). FLASH: Fast Length Adjustment of Short Reads to Improve Genome Assemblies. Bioinf. (Oxford England) 27 (21), 2957–2963. 10.1093/bioinformatics/btr507 PMC3198573 21903629

[B21] Martinsjunior P. A. Vieiraandrade R. G. Correafaria P. Oliveiraferreira F. Marques L. S. Ramosjorge M. L. (2013). Impact of Early Childhood Caries on the Oral Health-Related Quality of Life of Preschool Children and Their Parents. Caries Res. 47 (3), 211–218. 10.1159/000345534 23257929

[B22] Nyvad B. Crielaard W. Mira A. Takahashi N. Beighton D. (2013). Dental Caries From a Molecular Microbiological Perspective. Caries Res. 47 (2), 89–102. 10.1159/000345367 23207320

[B23] Obata J. Takeshita T. Shibata Y. Yamanaka W. Unemori M. Akamine A.. (2014). Identification of the Microbiota in Carious Dentin Lesions Using 16S rRNA Gene Sequencing. PloS One 9 (8). 10.1371/journal.pone.0103712 PMC4118920 25083880

[B24] Word Health Organization (2013). Oral Health Surveys: Basic Methods (Geneva: World Health Organization).

[B25] Paster B. J. Olsen I. Aas J. A. Dewhirst F. E. (2006). The Breadth of Bacterial Diversity in the Human Periodontal Pocket and Other Oral Sites. Periodontology 2000 42 (1), 80–87. 10.1111/j.1600-0757.2006.00174.x 16930307

[B26] Pitts N. B. Zero D. T. Marsh P. D. Ekstrand K. Weintraub J. A. Ramos-Gomez F.. (2017). Dental Caries. Nat. Rev. Dis. Primers 3 (1), 1–16. 10.1038/nrdp.2017.30 28540937

[B27] Priyadarshini H. R. Hiremath S. S. Fernandes B. (2013). Association Between Maternal - Child Levels of Salivary Mutans Streptococci and Early Childhood Caries. Dental Res. J. 10 (6), 728. 10.4103/1735-3327.122466 PMC3872622 24379859

[B28] Quast C. Pruesse E. Yilmaz P. Gerken J. Schweer T. Yarza P.. (2012). The SILVA Ribosomal RNA Gene Database Project: Improved Data Processing and Web-Based Tools. Nucleic Acids Res. 41 (D1), D590–D596. 10.1093/nar/gks1219 23193283 PMC3531112

[B29] Ramadugu K. Bhaumik D. Luo T. Gicquelais R. E. Lee K. H. Stafford E. B.. (2021). Maternal Oral Health Influences Infant Salivary Microbiome. J. Dental Res. 100 (1), 58–65. 10.1177/0022034520947665 PMC7755948 32859139

[B30] Shannon C. E. (1948). A mathematical theory of communication. Bell Syst. Tech. J. 27 (4), 379–423. 10.1002/j.1538-7305.1948.tb00917.x

[B31] Silva M. L. O. De Sousa ACDSB (2000). Health Care Practices in Family Context: A Comparative Case Study. Psicologia reflexao E Critica 13 (1), 0. 10.1590/S0102-79722000000300012

[B32] Simpson E. H. (1949). Measurement of Diversity. Nature 163 (4148), 688–688. 10.1038/163688a0

[B33] Simpson K. T. Thomas J. G. (2016). Oral Microbiome: Contributions to Local and Systemic Infections. Curr. Oral. Health Rep. 3 (1), 45–55. 10.1007/s40496-016-0079-x

[B34] Socransky S. Haffajee A. Cugini M. Smith C. Kent R. (1998). Microbial Complexes in Subgingival Plaque. J. Clin. Periodontol. 25 (2), 134–144. 10.1111/j.1600-051X.1998.tb02419.x 9495612

[B35] Subramaniam P. Suresh R. (2019). Streptococcus Mutans Strains in Mother-Child Pairs of Children With Early Childhood Caries. J. Clin. Pediatr. Dent. 43 (4), 252–256. 10.17796/1053-4625-43.4.5 31094631

[B36] Tanner A. C. R. Mathney J. M. Kent R. Chalmers N. I. Hughes C. V. Loo C. Y.. (2011). Cultivable Anaerobic Microbiota of Severe Early Childhood Caries. J. Clin. Microbiol. 49 (4), 1464–1474. 10.1128/JCM.02427-10 21289150 PMC3122858

[B37] Tao D. Li F. Feng X. Wong M. C. M. Lu H. (2018). Plaque Biofilm Microbial Diversity in Infants Aged 12 Months and Their Mothers With or Without Dental Caries: A Pilot Study. BMC Oral. Health 18 (1), 1–12. 10.1186/s12903-018-0699-8 30594172 PMC6311051

[B38] Teanpaisan R. Chaethong W. Piwat S. Thitasomakul S. (2012). Vertical Transmission of Mutans Streptococci and Lactobacillus in Thai Families. Pediatr. Dent. 34 (2), e24–e29. 22583873

[B39] Vos T. Abajobir A. A. Abate K. H. Abbafati C. Abbas K. M. Abd-Allah F.. (2017). Global, Regional, and National Incidence, Prevalence, and Years Lived With Disability for 328 Diseases and Injuries for 195 Countries 1990-2016: A Systematic Analysis for the Global Burden of Disease Study 2016. Lancet (London England) 390 (10100), 1211–1259. 10.1016/S0140-6736(17)32154-2 PMC5605509 28919117

[B40] Wakaguri S. Aida J. Osaka K. Morita M. Ando Y. (2011). Association Between Caregiver Behaviours to Prevent Vertical Transmission and Dental Caries in Their 3-Year-Old Children. Caries Res. 45 (3), 281–286. 10.1159/000327211 21576961

[B41] Wang Q. Garrity G. M. Tiedje J. M. Cole J. R. (2007). Naive Bayesian Classifier for Rapid Assignment of rRNA Sequences Into the New Bacterial Taxonomy. Appl. Environ. Microbiol. 73 (16), 5261–5267. 10.1128/AEM.00062-07 17586664 PMC1950982

[B42] Wang X. Shaffer J. R. Weyant R. J. Cuenco K. T. DeSensi R. S. Crout R.. (2010). Genes and Their Effects on Dental Caries May Differ Between Primary and Permanent Dentitions. Caries Res. 44 (3), 277–284. 10.1159/000314676 20516689 PMC2919434

[B43] Xu H. Hao W. Zhou Q. Wang W. Xia Z. Liu C.. (2014). Plaque Bacterial Microbiome Diversity in Children Younger Than 30 Months With or Without Caries Prior to Eruption of Second Primary Molars. PloS One 9 (2). 10.1371/journal.pone.0089269 PMC3938432 24586647

[B44] Yang C. M. C. K. (1993). Stopping Rules and Estimation for Recapture Debugging With Unequal Failure Rates. Biometrika 80 (1), 193–201. 10.1093/biomet/80.1.193

[B45] Zarco M. F. Vess T. J. Ginsburg G. S. (2012). The Oral Microbiome in Health and Disease and the Potential Impact on Personalized Dental Medicine. Oral. Dis. 18 (2), 109–120. 10.1111/j.1601-0825.2011.01851.x 21902769

[B46] Zaura E. Keijser B. J. F. Huse S. M. Crielaard W. (2009). Defining the Healthy “Core Microbiome” of Oral Microbial Communities. BMC Microbiol. 9 (1), 259–259. 10.1186/1471-2180-9-259 20003481 PMC2805672

